# Assessing the Sensitivity of EEG-Based Frequency-Tagging as a Metric for Statistical Learning

**DOI:** 10.1162/nol_a_00061

**Published:** 2022-02-16

**Authors:** Danna Pinto, Anat Prior, Elana Zion Golumbic

**Affiliations:** The Leslie and Susan Gonda Multidisciplinary Brain Research Center, Bar Ilan University, Ramat Gan, Israel; Department of Learning Disabilities, University of Haifa, Haifa, Israel

**Keywords:** auditory statistical learning, frequency-tagging, EEG

## Abstract

Statistical learning (SL) is hypothesized to play an important role in language development. However, the measures typically used to assess SL, particularly at the level of individual participants, are largely indirect and have low sensitivity. Recently, a neural metric based on frequency-tagging has been proposed as an alternative measure for studying SL. We tested the sensitivity of frequency-tagging measures for studying SL in individual participants in an artificial language paradigm, using non-invasive electroencephalograph (EEG) recordings of neural activity in humans. Importantly, we used carefully constructed controls to address potential acoustic confounds of the frequency-tagging approach, and compared the sensitivity of EEG-based metrics to both explicit and implicit behavioral tests of SL. Group-level results confirm that frequency-tagging can provide a robust indication of SL for an artificial language, above and beyond potential acoustic confounds. However, this metric had very low sensitivity at the level of individual participants, with significant effects found only in 30% of participants. Comparison of the neural metric to previously established behavioral measures for assessing SL showed a significant yet weak correspondence with performance on an implicit task, which was above-chance in 70% of participants, but no correspondence with the more common explicit 2-alternative forced-choice task, where performance did not exceed chance-level. Given the proposed ubiquitous nature of SL, our results highlight some of the operational and methodological challenges of obtaining robust metrics for assessing SL, as well as the potential confounds that should be taken into account when using the frequency-tagging approach in EEG studies.

## INTRODUCTION

*Statistical learning* (SL) refers to the remarkable ability to implicitly learn the rules and relationship between different stimuli and events in the environment. The capacity for SL has been studied in both humans and non-human species (Kang et al., 2021; Santolin et al., 2016; Santolin & Saffran, 2018), and has been demonstrated across different sensory domains, emerging relatively early in infancy (Gómez & Gerken, 2000; Graf Estes et al., 2007; Pelucchi et al., 2009; Saffran et al., 1996, 1997). SL has been hypothesized to play an important role in the development of many key cognitive abilities such as communication skills, object recognition, and sensory-motor learning (Arciuli & von Koss Torkildsen, 2012; Emberson et al., 2011; Erickson & Thiessen, 2015; Evans et al., 2009; Hsu et al., 2014; Kent & Read, 2002; Kidd, 2012; Kidd & Arciuli, 2016; Misyak & Christiansen, 2012; Siegelman et al., 2017; Spencer et al., 2015; Thiessen & Saffran, 2003, 2007). And yet, despite the potentially pivotal role of SL for cognition, current empirical metrics used to assess SL, particularly at the level of individuals, are largely indirect, and often have low sensitivity.

In typical SL experiments a sequence of stimuli is presented in which the transitional probabilities between consecutive stimuli are manipulated so that some items carry predictive information about which stimulus will follow. One prominent example is the artificial language paradigm, where participants hear sequences of syllable triplets that are always presented consecutively (transitional probability = 1) and thus form words in an artificial language (which we refer to throughout this paper as *pseudowords*). Participants are exposed to these stimuli for a short period of time (exposure phase), which can range between 2 and 24 min (Batterink et al., 2015; Franco, Gaillard, et al., 2015; Karuza et al., 2013; Saffran et al., 1997), and then perform a test to assess whether the statistical regularities within the sequence have been picked up by the listener. A variety of explicit and implicit tests can be applied to evaluate SL following an exposure phase, such as a 2-alternative forced-choice test (2AFC) or a target-detection task (Batterink, 2017; Batterink & Paller, 2017; Batterink et al., 2015). Behavioral results on these tests usually show moderate yet above-chance performance when analyzed at the group-level. For example, performance on 2AFC tasks ranges between 54% and 68% across studies, which constitutes a significant yet fairly weak demonstration of learning (Batterink & Paller, 2017; Batterink et al., 2015; Buiatti et al., 2009; de Diego-Balaguer et al., 2015; Fernandes et al., 2010; Franco et al., 2011; Franco, Gaillard, et al., 2015; Frost et al., 2015; Kim et al., 2009; Olson & Chun, 2001; Saffran et al., 1997; Siegelman & Frost, 2015; Toro et al., 2005; Turk-Browne et al., 2005; Tyler & Cutler, 2009). However, success rates of individual participants are rarely reported, and the few studies that do include this data find that at least 30% of the participants show no evidence for SL at all and in many individuals behavioral effects are quite small (Cunillera et al., 2008; Franco, Gaillard, et al., 2015; Romberg & Saffran, 2013). It is also worth noting that the within-subject correlation between different behavioral tasks (e.g., explicit vs. implicit tests) is often low, raising questions about the optimal experimental operationalization for capturing and assessing SL (Batterink et al., 2015; Franco, Eberlen, et al., 2015; Misyak et al., 2010). Given the hypothesized fundamental role of SL for a variety of cognitive processes (Arciuli, 2017; Erickson & Thiessen, 2015), it seems pertinent to develop a more robust empirical measure of SL, that can reliably assess whether or not SL has occurred at the level of individual subjects.

Rather than relying on post-exposure behavioral testing for assessing SL, an alternative approach is to analyze participants’ neural activity during the exposure phase and look for evidence that statistical regularities within the stimulus are being picked up. Along these lines, an EEG-based frequency-tagging approach has recently been proposed using a variation of the artificial language paradigm (Batterink, 2020; Batterink & Paller, 2017, 2019; Buiatti et al., 2009; Choi et al., 2020; Elmer et al., 2021; Getz et al., 2018; Henin et al., 2021; Kiai & Melloni, 2021; Lukics & Lukács, 2021). In this version, syllables are presented at a constant rate (e.g., *X Hz*), and consequently the tri-syllabic pseudowords also occur at a fixed rate (*X/3 Hz*). These two levels of information are thus distinguishable in frequency, which can potentially be observed in the spectrum of the EEG neural recording. This frequency-tagging approach has been successfully employed for studying real speech processing, demonstrating that a peak at the word-level frequency emerges in the spectrum of the neural response when syllables make up words that participants know, but not if they are in a foreign language or do not form recognizable words or phrases (Ding et al., 2016; Lu et al., 2021; Luo & Ding, 2020; Makov et al., 2017; Sheng et al., 2019). Applying this approach to a SL paradigm, Batterink and Paller (2017) demonstrated that the ratio between the power at the syllable vs. pseudoword frequency during the exposure-phase was positively correlated with behavioral performance on an implicit (but not an explicit) behavioral task for assessing SL. This was taken as an indication for the adeptness (and perhaps advantage) of using frequency-tagging to assess SL experimentally, circumventing the need for overt post-exposure behavioral testing.

However, despite the promise held by this approach as providing a more direct and objective measure of SL, some of the previous findings raise questions regarding the sensitivity of this measure, particularly at the level of individual subjects. For example, the individual-level data presented by Batterink and Paller (2017) indicate that SL effects were limited only to a subset of participants, with others showing effects in the opposite direction. Moreover, in that study significant effects were also reported when participants listened to random sequences of syllables, where there should not be any SL. As suggested by recent studies, these results may have been somewhat confounded by acoustic contributions to the neural response at the pseudoword frequency that occur naturally for this type of stimuli (Luo & Ding, 2020; van der Wulp, 2021). In particular, in a recent re-analysis of the EEG data originally reported by Batterink and Paller (2017), van der Wulp (2021) demonstrated that at least some of the reported effects can be explained by variations in place of articulation of different syllables (known as the *obligatory contour principle*; OCP), rather than by SL of transitional probabilities between syllables. Consequently, without proper controls, the magnitude of the neural response at the pseudoword frequency might be over-interpreted as only reflecting SL, while the acoustic contribution to this peak is discounted or ignored.

Therefore, it seems that further validation of the frequency-tagging approach is required, and adequate controls implemented, before adopting it as a demonstrably preferable measure of SL. This is an important endeavor not only for furthering our understanding of the potential for, and possible limitations of, frequency-tagging for studying SL in humans, but also for assessing its potential sensitivity for use in clinical conditions (e.g., non-consciousness states; Gui et al., 2020; Sokoliuk et al., 2021) as well as in non-human species, where data analysis typically relies on within-subject effects and not on group-effects.

## MATERIALS AND METHODS

### Participants

Participants were 40 adults (25 female, 35 right-handed), ages 20–38 (mean = 24.78, *SD* = 3.96). Due to technical issues, EEG data from one participant and behavioral data on the implicit test from 13 participants were lost. All participants reported normal hearing and had no history of psychiatric or neurological disorders and were native Hebrew speakers. They were paid or received course credit for participation. The study was approved by the IRB committee at Bar Ilan University and participants read and signed an informed consent form prior to starting the experiment.

### EEG Recording and Apparatus

EEG was recorded using a 64 Active-Two system (BioSemi) with Ag-AgCl electrodes, placed according to the 10–20 system, at a sampling rate of 1024 Hz. Additional external electrodes were used to record from the mastoids bilaterally, and both vertical and horizontal electrooculography electrodes were used to monitor eye movements. The experiment was conducted in a dimly lit, acoustically and electrically shielded booth. Participants were seated on a comfortable chair and were instructed to keep as still as possible and breathe and blink naturally. Experiments were programmed and presented to participants using PsychoPy (https://www.psychopy.org; Peirce et al., 2019). Visual instructions were presented on a computer monitor, and auditory stimuli were delivered through in-ear earphones (Etymotic ER-1). Button-press responses were recorded using a serial response-box (Cedrus RB).

### Stimuli

**Figure 1.**
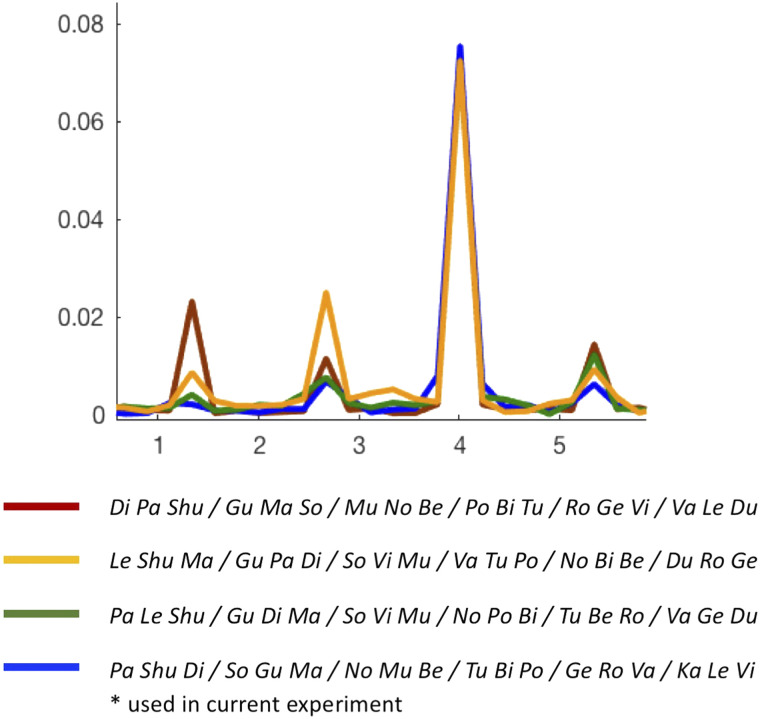
Modulation spectrum for four different versions of the artificial language stimuli, all composed of similar syllables but combined to form different pseudowords. As expected, all stimuli contain a peak at the 4-Hz syllable rate. However, as shown here, additional peaks are observed at the pseudoword rate (1.3 Hz) and its harmonics, and the magnitude of these peaks varies for the different combinations. As shown in similar studies (Har-shai Yahav & Zion Golumbic, 2021; Luo & Ding, 2020; van der Wulp, 2021), these peaks stem from the fact that the same subset of syllables is present in constant positions within the stimulus streams. The artificial language stimuli chosen in the current experiment was a combination of syllables that generated relatively small peaks at pseudoword rate frequencies and its harmonics in the modulation spectrum; however, these were nonetheless still present (blue line). This motivated the use of position-controlled stimuli as a means to control for these inherent acoustic peaks, which has a modulation spectrum similar to the artificial language stimuli. This allowed us to attribute significant differences in the neural response between these two stimuli to effects of statistical learning, rather than trivial differences in their acoustic structure.

The stimuli consisted of 18 CV syllables recorded in a male voice. Individual syllables were recorded in random order to avoid effects of coarticulation, and only recordings with a flat intonation were used. The recordings were edited offline so that each syllable was precisely 250 ms long (silence periods were added if necessary), and their loudness was equated (Audacity software; https://www.audacityteam.org/). Additional audio-editing and concatenation of syllables into longer streams were performed in Matlab (Mathworks; https://www.mathworks.com/). The artificial language consisted of six tri-syllabic pseudowords (*PaShuDi*, *SoGuMa*, *NoMuBe*, *TuBiPo*, *GeRoVa*, *KaLeVi*), with each syllable appearing in only one pseudoword. Accordingly, the within-word transitional probability was 1 and the between-words transitional probability was 0.2. Given that the modulation spectra of this type of stimuli naturally contains acoustic-driven peaks and frequencies besides the syllable rate itself (Luo & Ding, 2020; van der Wulp, 2021), we tested the modulation spectrum of several syllable-triplet combinations and selected the combination that yielded the smallest peaks at the pseudoword rate and/or its harmonics as the pseudowords in this experiment (Figure 1). We also confirmed that the pseudowords do not sound similar to known Hebrew or English words.

**Figure 2.**
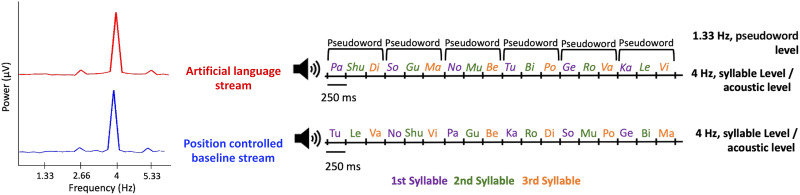
Diagram illustrating structure of the artificial language and position-controlled baseline streams used in the current experiment. Left: The modulation spectrum of the artificial language stream (red) and the position-controlled baseline stream (blue). Both show a prominent peak at the syllable rate (4 Hz), as well as more modest peaks at 2.66 Hz and 5.33 Hz, which are the first and third harmonics of the triplet rate. Right: Examples of the auditory streams. Both stimuli were composed of the same syllables, presented at a constant rate of 4 Hz. Each stream consisted of syllable triplets, with each syllable consistently either at the 1st (blue), 2nd (green), or 3rd (orange) position. In the artificial language stream, fixed syllable triplets were used in forming pseudowords (within-pseudoword transitional probability = 1; between-pseudoword transitional probability = 0.2), whereas in the position-controlled baseline stream all possible triplet combinations were used, resulting in a consistent transitional probability of 0.2 between all syllables.

Since the acoustic-driven contributions to the modulations spectrum at the triplet-related frequencies could not be fully eliminated from the artificial language stimulus, we constructed a position-controlled baseline stimulus to estimate the extent of these acoustic contributions to the neural signal. The baseline stimulus consisted of syllable triplets constructed from the same 18 syllables, but with less consistent transitional probabilities between them. Similar to the approach used by Makov et al. (2017), in these position-controlled syllable triplets each syllable maintained the position it held in the original pseudowords; however, all possible combinations were allowed (Figure 2, right). This yielded a constant transitional probability of 0.2 both within-triplet and between-triplets in the baseline stimulus.

Both the pseudowords and the position-controlled triplets were concatenated to create three 3.22-min-long streams of the artificial language and baseline conditions. The order of pseudowords and position-controlled triplets in each stream was pseudorandomized to avoid immediate repetitions of the same triplet and ensure their equal distribution over time. Comparison of the modulation spectra confirmed that this approach resulted in similar peaks at 1.33 Hz for both the artificial language and baseline streams, making them highly comparable acoustically and allowing us to gauge the effect of within-word transitional probabilities on the 1.33 Hz peak in the neural response, above and beyond any potential acoustic contributions from the stimulus itself (Figure 2, left). Both the artificial language and baseline streams included a 5 sec ramping up/down period, to avoid inadvertent cues about syllable positions or pseudoword boundaries.

### Experimental Procedure

#### Exposure phase

The experiment consisted of several stages. It started with a baseline exposure stage during which participants listened to the baseline condition streams of concatenated syllables described above. These consisted of hearing three 3.22-min-long streams (separate blocks, with breaks between them; total exposure time: ∼10 min). Participants were instructed to listen passively to the stimuli with their eyes open and fixated on a point on the screen. In this stage no additional instructions were given. After a brief break they were exposed to the *three blocks* of the artificial language streams. Here participants were explicitly told that the streams are made up of words in an artificial language, which they are requested to learn for subsequent testing. However, participants were not told the length or number of the pseudowords. The order between exposure phases was held constant to avoid carryover learning effects in the baseline condition after exposure to the artificial language.

During the break between the baseline and artificial language conditions, participants performed an English vocabulary task. This task was chosen as a way to clear their verbal working memory and also in order to test the hypothesis that statistical learning abilities are correlated with second language learning abilities (since all our participants learned English as a second language in school). Unfortunately, the results of the vocabulary test from almost half of the participants were lost due to technical difficulties, which did not allow us to further explore this research question in the current study.

#### Testing phase

The testing phase consisted of two behavioral tests:

##### 2-Alternative forced choice task (2AFC).

The explicit 2AFC discrimination task was designed to follow the commonly used procedure for explicit testing of statistical learning (Batterink & Paller, 2017; Batterink et al., 2015; Buiatti et al., 2009; Fernandes et al., 2010; Franco et al., 2011; Franco, Gaillard, et al., 2015; Saffran et al., 1997; Toro et al., 2005, 2011; Tyler & Cutler, 2009; Wang & Saffran, 2014). In addition to the six pseudowords that made up the artificial language, six additional *part-words* were created consisting either of the last two syllables of one pseudoword combined with the first syllable of another, or the last syllable of one pseudoword combined with the first two syllables of another. As such, these are combinations that participants could have heard occasionally during the learning phase, but not as frequently as the actual pseudowords. In each trial, one pseudoword and one part-word were played (random order), and participants were required to indicate via button-press which one was familiar to them from the artificial language learning phase. This test consisted of a total of 36 trials (all possible pairs of pseudowords and part-words).

**Figure 3.**
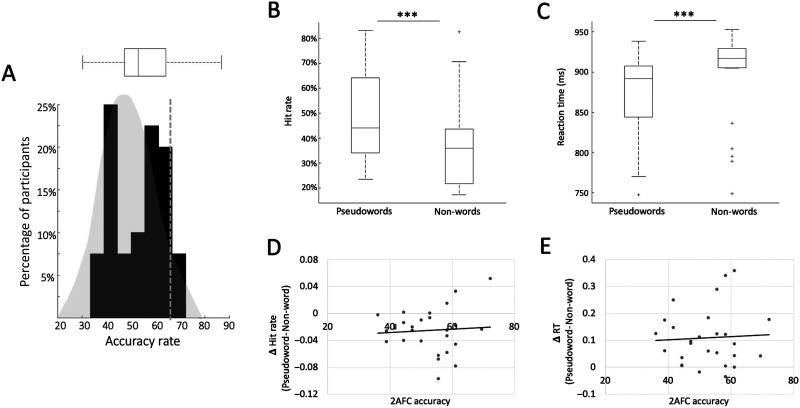
Behavioral results. (A) 2AFC results: Histogram of accuracy rates on the 2AFC task across all participants (black), overlaid on the background of the a priori binomial distribution of chance-level results in the current design (gray). Top: Interquartile range and group median of 2AFC results. Dashed gray line: the *p* = 0.05 cutoff for determining whether individual level performance was significantly above chance (relative to the null distribution). (B&C) Target detection results: Interquartile range and group-level median for hit rates and reaction times (RTs) in response to target syllables that occurred in the 3rd position of pseudowords vs. targets that occurred in non-words. For both metrics, performance was improved for targets within pseudowords, as indicated by the asterisks (*p* < 0.001) between the conditions. Outliers are indicated by a gray plus (+) symbol. (D&E) Scatterplots depicting the within-subject relationship between performance on the 2AFC task and the target detection task. No significant correlation was found between accuracy on the 2AFC task and the magnitude of the behavioral effects in the target detection task (difference in hit rate / RTs for targets in pseudowords vs. targets in non-words).

Group-level statistical analysis of performance on the 2AFC task consisted of a single-sample *t* test testing whether accuracy rates were significantly higher than chance (50%), as commonly done in similar studies (Batterink & Paller, 2017; Batterink et al., 2015; Buiatti et al., 2009; Fernandes et al., 2010; Franco et al., 2011; Franco, Gaillard, et al., 2015; Saffran et al., 1997; Toro et al., 2005, 2011; Tyler & Cutler, 2009; Wang & Saffran, 2014). However, since the 2AFC task consists of only 36 trials and does not necessarily meet the assumptions required for a *t* test, we further simulated the null distribution of our specific experiment using a permutation test. We simulated a random 2AFC guessing pattern for 36 trials and calculated the “random hit rate” of that simulation. This procedure was repeated 1,000 times, producing a null distribution reflecting the probability of achieving a particular hit rate “by chance” (Figure 3A, shown in gray). Furthermore, we assessed the significance of performance in individual participants by comparing their accuracy rates to a binomial distribution for 36 2AFC trials (Franco, Gaillard, et al., 2015; Siegelman et al., 2017), allowing us to establish which participants showed evidence for statistical learning according to the 2AFC test.

##### Target detection task.

The explicit test was followed by an implicit target detection task, designed based on previous studies using a similar approach (Batterink et al., 2015; Batterink & Paller, 2017). In each trial one syllable was designated as the target and was played twice for participants to familiarize themselves with the sound (e.g., ***Va***). Then a sequence of syllables was played, and participants were required to press a button when they heard the target syllable. The sequences contained pseudowords from the artificial language as well as other triplet-syllable combinations (non-words). The target syllable in each trial (e.g., ***Va***) was placed strategically within the sequences and could occur either as the 3rd syllable of a pseudoword presented in the exposure phase (e.g., *GeRo**Va***) or as the 1st or 3rd syllable in a non-word (e.g., ***Va**ShuPo* or *PaMu**Va***). In this task, enhanced target detection performance for targets presented as the 3rd syllable of a previously learned pseudoword (vs. syllables in a non-word) would serve as an indication that participants had successfully learned the structure of the artificial language because they are able to anticipate the target syllable.

For this task, syllables were presented at a constant rate of 2 Hz with each trial lasting 22.5 sec and including 4–8 targets. The entire task consisted of 24 trials (4 trials per target syllable). A button-press was considered a *hit* if it fell within 1 sec after the presentation of a target syllable. Otherwise, it was considered a *false alarm*. The order of the explicit 2AFC task and the implicit target detection task was kept constant and not randomized across participants. Since the 2AFC task is the more common test for SL, we felt it was important to administer it immediately after the exposure phase, and to avoid its potential contamination by exposure to additional syllable sequences in the implicit task.

Group-level statistical analysis consisted of paired *t* tests of hit rates and reaction times (RTs) for targets occurring within pseudowords vs. non-words (responses to targets occurring in 1st and 3rd position of non-words were grouped together, since we found no differences between them). Statistical analysis at the level of individual participants was conducted using permutation tests. The permutation test consisted of random relabeling of all the responses of a particular participant into two random conditions, regardless of their original status as pseudoword/non-word targets, and taking the difference between the means of the two random conditions. This procedure was repeated 1,000 times, and the differences of the means extracted from each permutation were used to form a null distribution for each participant. We then took the real difference between the pseudoword and non-word targets in the original data and compared it to the null distribution. The difference between conditions was considered significant if the real value fell in the top fifth percentile of the null distribution (one-tailed). This procedure was performed for both accuracy and RT data.

We further tested whether performance on the two behavioral tasks was correlated, by calculating the Pearson correlations between explicit 2AFC accuracy rates and the implicit target detection task (differences in hit rates / RTs for targets occurring within pseudowords vs. non-words).

### EEG Data Analysis

#### Preprocessing and spectral analysis

EEG data were measured only during the exposure phase of the experiment, and were not measured during the testing phase. Data from three blocks (∼11 min) of both conditions were preprocessed and cleaned together. All EEG preprocessing and analysis were performed in Matlab (The Mathworks) using the toolbox FieldTrip (Oostenveld et al., 2011) as well as custom written scripts. Raw data were first visually inspected and gross artifacts that exceeded ±50 μV (and were not eye movements) were removed. Then, independent component analysis was performed to identify and remove components associated with horizonal or vertical eye movements as well as heartbeats. Any additional noisy electrodes / segments of the data that remained after this procedure, and that exhibited either extreme high-frequency activity (>40 Hz) or low-frequency activity/drifts (<1 Hz), were either replaced with the weighted average of their neighbors using an interpolation procedure, or (if that was not possible) removed.

The clean data were analyzed separately for the baseline and artificial language exposure blocks. The continuous data were segmented into 4.5 sec epochs, which correspond to 6 syllable triplets. Critically, these segments were perfectly aligned such that they all started with the onset of a triplet. Inter-trial phase coherence (ITPC) was used to analyze the neural response at specific frequencies. ITPC was calculated as follows: The fast Fourier transform was calculated for each individual segment between 0.3 and 6 Hz using a Hanning window. The phase component at each frequency was used to calculate the ITPC, which is the sum (absolute value) of the phases across segments, as follows:

$$\text{ITPC}=\frac{1}{N}\left|\sum_{k=1}^{N}e^{i*\phi_{k}}\right|$$

ITPC analysis was performed separately for the baseline and artificial language exposure blocks and was calculated across all blocks as well as separately for each of the three exposure blocks per condition.

Statistical analysis of EEG data focused a priori on four frequencies of interest: the syllable presentation rate (4 Hz); the pseudoword rate (1.33 Hz); and two harmonics of the pseudoword frequency (2.66 Hz and 5.33 Hz). We tested for differences between the baseline and artificial language conditions at each of these frequencies, both at the group level and as well at the level of individual subjects.

#### Group-level analysis

To avoid a priori assumptions as to which electrodes would show an effect of statistical learning, we performed statistical tests both on the average ITPC across all electrodes (one-way paired *t* test and dependent-sample Bayesian analysis; JASP Team, 2019), and at each electrode individually (*cluster-based* correction for multiple comparisons). Based on previous research (Batterink & Paller, 2017, 2019; Buiatti et al., 2009), we hypothesized that finding significant peaks at 1.33 Hz and/or its harmonics 2.66 Hz and 5.33 Hz in the artificial language condition would serve as an indication that the statistical regularities within the artificial language had indeed been identified and the stream was parsed correctly into tri-syllabic pseudowords. In these analyses, the direction of the expected results was determined a priori based on the results of previous findings (Batterink & Paller, 2017, 2019; Buiatti et al., 2009), predicting increased responses in the artificial language vs. baseline condition at frequencies associated with the pseudoword rate and its harmonics (1.33 Hz, 2.66 Hz, and 5.33 Hz), and the reverse pattern at the syllable rate (4 Hz). Since these hypotheses were determined a priori we did not apply further corrections for multiple corrections of the four frequencies of interest.

We additionally tested whether ITPC changed over the course of the exposure stage, using a linear regression analysis. For this we calculated the average ITPC across all electrodes separately for each of the three blocks in each exposure condition, and at each frequency of interest. Average ITPC values for each participant per block were fit using a linear regression model, as implemented in R’s glmer function (lme4 package; Bates et al., 2015), with condition (artificial language vs. baseline) and block (1–3) as fixed effects and participant as a random effect (model: ITPC = Condition * Block + (1|Participant)). Evidence for SL should manifest as a significant interaction between condition and block, indicating that ITPC increases systematically across blocks in the artificial language condition, but not in the baseline condition. We used Helmert forward contrast coding, which compares the level of each variable with the mean of the subsequent levels of that variable. The regression model was applied separately to the data at each frequency of interest.

#### Individual-level analysis

**Figure 4.**
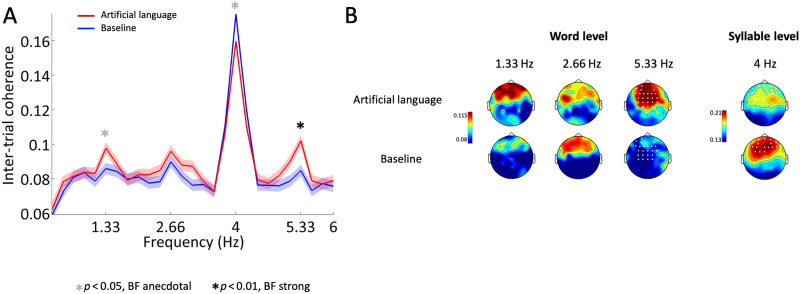
ITPC results. (A) Grand average of the ITPC spectrum in the artificial language (red) and baseline (blue) conditions, averaged across all electrodes. Shading indicates *SEM* across participants (*n* = 39). Asterisks indicate peaks where there was a significant difference between conditions at the group level (*t* test, *p* < 0.05), accompanied by either strong (black) or anecdotal (gray) BFs. (B) Scalp topographies of the ITPC response at each of the four frequencies of interest. White dots indicate electrodes where a significant difference was found between the artificial language and baseline conditions, when comparisons were performed separately at each electrode (cluster corrected).

One of our primary goals was to assess whether evidence for statistical learning can be gleaned at the level of individual participants using this frequency-tagging method. To achieve this, we performed two statistical analyses on the data from each participant. First, we used within-subject permutations to test for significant differences in ITPC between the baseline and artificial language conditions. For each participant, we randomly switched the label of half of the epochs between the baseline and artificial language conditions and calculated the ITPC difference between these two null conditions at each of the four frequencies of interest (averaged across all electrodes). This was repeated 1,000 times to create a null distribution: If the real difference in ITPC between conditions fell within the top 5% of this null distribution, it was considered significant (*p* = 0.05, one way). This procedure was performed for each participant at each frequency of interest. This analysis was performed on the average ITPC across all electrodes in order to avoid reducing our statistical power due to multiple comparisons. However, since averaging together this analysis might have also reduced the overall signal-to-noise ratio (SNR), we repeated the same analysis using only the subset of electrodes that were found to have a significant effect of SL in the group-level analysis (shown in Figure 4). Lastly, we also tested for correspondence between the EEG-based metric and the behavioral metrics.

## RESULTS

### Behavioral Results

Accuracy levels on the 2AFC task at the group level were not significantly different than chance [mean = 52.56%, *SD* = 0.10; *t*(38) = 1.60, *p* = 0.12]. Moreover, when comparing accuracy rates at the individual level to the a priori binomial distribution of accuracy rates expected by chance (Figure 3A), we found that only *n* = 3 participants (7.5%) had performance that fell into the top fifth percentile of the chance-level distribution (cutoff = 65%, *p* = 0.05).

Due to technical failures, data from the target detection task were available for only 27 of the 40 participants. In the remaining subset, we found significantly higher accuracy and faster RTs for target syllables that occurred in the 3rd position of a pseudoword vs. targets that were part of a non-word [hit rates: *t*(26) = 5.35, *p* < 0.001; RTs: *t*(26) = −4.05, *p* < 0.001] (Figure 3B&C). Statistical analysis of behavioral results at the individual level found that *n* = 12 participants had a significant effect on hit rate, and *n* = 9 participants had a significant effect on RTs. Overall, *n* = 18 participants (70%) had a significant effect on *either* hit rate or RT, but only *n* = 3 participants showed significant effects for *both* measures. When comparing individual level results on the two tasks within-participants, we failed to find any significant correlations between accuracy rates on the 2AFC task and the magnitude of the behavioral effects in the target detection task (difference in hit rate / RTs for targets in pseudowords vs. targets in non-words; hit rate: Pearson’s *r*^2^ = 0.05, *p* = 0.79; RTs: *r*^2^ = 0.07, *p* = 0.72; Figure 3D&E).

### EEG Results

#### Group-level analysis

Figure 4A shows the mean ITPC spectra across all participants and electrodes, across the two conditions. As expected, prominent peaks are observed at the syllable rate (4 Hz) and at the triplet rate (1.33 Hz), as well as at the 1st and 3rd harmonics (2.66 Hz and 5.33 Hz, respectively), in *both* the baseline and the artificial language conditions, indicating that the mere presence of a peak in the spectrum is not sufficient evidence for SL per se. The ITPC spectra from all individual participants in the baseline condition is shown in Figure S1 (see the Supporting Information at https://doi.org/10.1162/nol_a_00061), which illustrates the existence of these peaks in most individuals as well as the variance among them.

At the same time, when comparing the two conditions we do find effects that are consistent with previous effects of SL. The ITPC at the pseudoword rate (1.33 Hz) was significantly larger in the artificial language condition vs. the baseline [*t*(38) = 2.01, *p* = 0.023; BF = 2.057 (anecdotal support)], and an even stronger enhancement was found at the 3rd pseudoword rate harmonic [5.33 Hz; *t*(38) = 2.881, *p* = 0.003; BF = 11.893 (strongly supported)], although the peak at the 1st pseudoword rate harmonic (2.66 Hz) was not significantly different between conditions [*t*(38) = 0.804, *p* = 0.213; BF = 0.36 (moderate acceptance of H_0_)]. The ITCP at the syllable rate (4 Hz) was also modulated by the stimulus type but in the opposite direction, with a reduced peak in the artificial language condition vs. the baseline [*t*(38) = −1.858, *p* = 0.035; BF = 2.057 (anecdotal support)].

When repeating the statistical analysis at each electrode separately, we found significant clusters of electrodes at the 3rd pseudoword rate harmonic (5.33 Hz) and the syllable rate (4 Hz). The effect at the 3rd pseudoword rate harmonic frequency was observed in 25 mid-central electrodes, and the effect at the syllable rate frequency was observed in 17 mid-frontal electrodes as indicated in the topographic map in Figure 4.

#### By-block linear regression analysis

**Figure 5.**
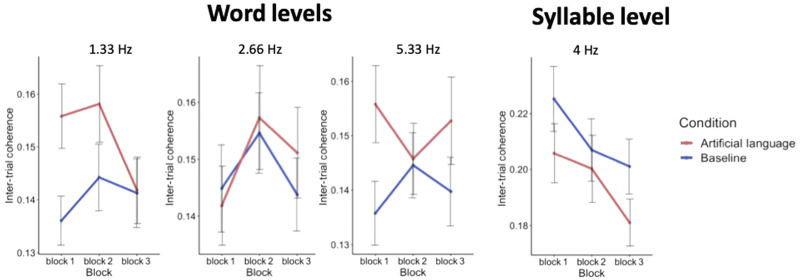
Results of by-block analysis. Mean ITPC across the three exposure blocks of each condition, averaged across all electrodes, at each of the frequencies of interest. Linear regression analysis confirmed the main effect of condition at 1.33 Hz and 5.33 Hz (artificial language condition > baseline), but neither the effects of block nor the interaction between condition and block were significant at any of the frequencies.

Besides looking at the ITPC across the entire experiment, we also performed a linear regression analysis to test for changes in the response across the three exposure blocks (Figure 5). The main effects of condition (artificial language vs. baseline), reported above for responses as 1.33 Hz and 5.33 Hz, were confirmed in this analysis as well [*F*(190) = 2.25, *p* = 0.03, and *F*(228) = 2.12, *p* = 0.03 respectively]. However, the effect of block and interactions between condition and block were not significant at any of the frequencies of interest (see Table 1 for full statistical results). Rather, all the effects of condition seem to be present already in the first exposure block and were not further enhanced over time.

**Table 1.** Summary of statistical results of block analysis. Significant results are indicated in bold and with an asterisk

Contrast	Frequency	β	*t*	*p*
Block number (1 vs. 2)	Word	−0.007	−0.88	0.38
	Syllable	0.02	1.98	0.05
	1st Harmonic	−0.004	−0.49	0.63
	3rd Harmonic	−0.006	−0.78	0.43
Block number (1 & 2 vs. 3)	Word	0.003	0.34	0.74
	Syllable	0.006	0.48	0.63
	1st Harmonic	0.01	1.06	0.29
	3rd Harmonic	0.005	0.51	0.61
Condition (artificial language vs. baseline)	Word	0.11	2.25	0.03*
	Syllable	−0.02	−2.15	0.03*
	1st Harmonic	0.002	0.40	0.69
	3rd Harmonic	0.01	2.10	0.04*
Interaction Block number (1 vs. 2) × Condition	Word	0.01	1.163	0.25
	Syllable	−0.006	−0.40	0.69
	1st Harmonic	0.008	−0.64	0.52
	3rd Harmonic	0.01	1.12	0.26
Interaction Block number (1 & 2 vs. 3) × Condition	Word	0.01	1.07	0.28
	Syllable	0.01	0.76	0.45
	1st Harmonic	−0.005	−0.32	0.75
	3rd Harmonic	−0.01	−0.88	0.38

#### Individual participant analysis

**Figure 6.**
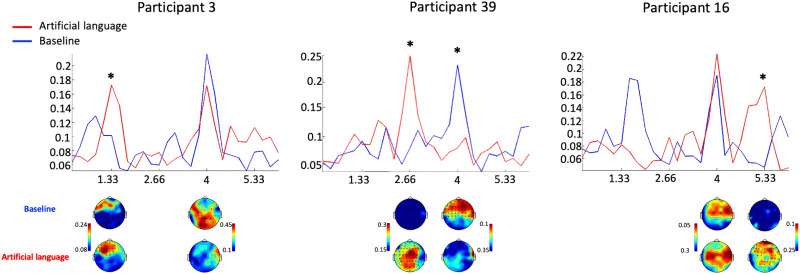
Examples of ITPC spectral of individual participants. ITPC spectra in the artificial language (red) and baseline (blue) conditions from three participants who showed significant differences between the artificial language and baseline conditions, albeit at different pseudoword-related frequencies (indicated by an asterisk). Spectra from each participant are shown from the average across all electrodes.

Assessment of SL effects from the neural response at the level of individual participants was conducted using permutation tests. Since SL effects could potentially manifest either at the pseudoword rate itself or at any of its harmonics, this analysis was performed at all the frequencies of interest. When performing the statistical analysis on the average ITPC across all electrodes, we found significant effects of condition in 12/39 participants (31%), with larger responses in the artificial language condition vs. the baseline. Of these participants, in *n* = 5 the effects were at 1.33 Hz, *n* = 3 at 2.66 Hz, and in *n* = 4 at 5.33 Hz. Only one participant had significant effects at more than one frequency. In addition, the reduced response in the artificial language condition at the syllable rate (4 Hz) that was observed at the group level was found to be significant for *n* = 6 participants. For specific examples of the ITPC spectrum of individual participants who showed significant results see Figure 6. When repeating the analysis using only the electrodes that had significant SL effects at the group level (shown in Figure 4), we found similar results: 13/39 (33%) participants had a significant effect of condition, with larger responses for the artificial language condition than the baseline. Of them, in *n* = 9 the effects were at 1.33 Hz, in *n* = 3 at 2.66 Hz, and in *n* = 5 at 5.33 Hz. Three participants had significant effects at more than one frequency. Also, the reduced response in the artificial language condition at 4 Hz was found to be significant in *n* = 7 participants. We note that the latter analysis is a little circular (selection of electrodes based on a previous group-level result). However, the convergence of results in these two analyses (one more conservative, one more permissive) supports the overall conclusion of a low prevalence of SL effects in individual participants.

#### Correspondence between ITPC effects and behavior

We next tested whether the ITPC response to pseudoword rates in the artificial language condition corresponds to performance on behavioral tasks administered post-exposure. Since the individual level analysis revealed inconsistencies in the specific pseudoword-related frequencies where significant differences were found across participants (i.e., at the pseudoword frequency itself or one of the harmonics), this prevented us from performing a simple correlation analysis between the ITPC at a particular frequency and behavioral measures. To overcome this between-participant variability, we used two different approaches.

First, we took the average ITPC in the artificial language condition across the three pseudoword-related frequencies (1.33 Hz, 2.66 Hz, and 5.33 Hz), and calculated the Pearson correlations with each behavioral measure. This did not, however, yield any significant results (correlation with 2AFC accuracy: *r*^2^ = 0.16, *p* = 0.33; correlation with target detection hit rate: *r*^2^ = 0.13, *p* = 0.50; correlation with target detection RTs: *r*^2^ = −0.05, *p* = 0.81).

**Figure 7.**
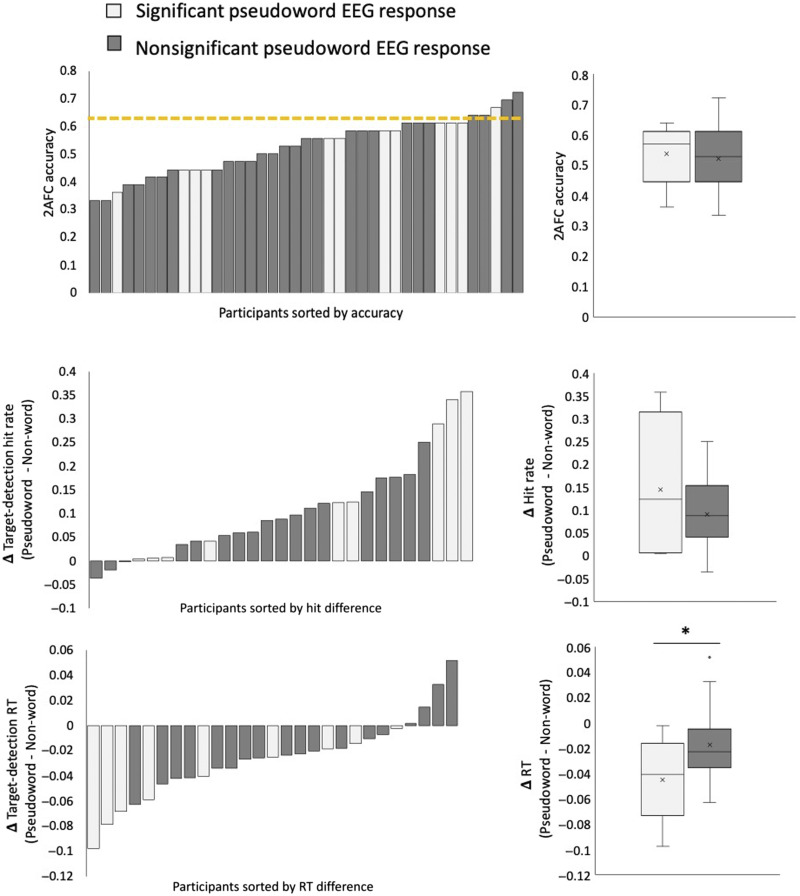
Correspondence between and pseudoword EEG response during the exposure period and post-exposure behavioral tasks. Participants were divided into two groups based on whether a significant response to pseudowords was found in their neural response during the exposure period (gray) or not (black). The left-hand panels show the results of all participants on each of the three behavioral measures: 2AFC accuracy, effects size on hit rate, and RTs in the target detection task (sorted by effect size and color-coded by group). The right-hand panels show the means and *SEM* of these behavioral measures, in each group. A significant difference between-groups was found for the target detection RT effect (bottom panel, *p* < 0.05), but not for the other behavioral measures.

Second, we separated the participants into two groups based on whether there was evidence for SL from their neural data (regardless of the frequency where this effect was observed) and compared the behavioral results between the two groups. We used the Welch’s test for unequal variance to account for the different sample sizes in the two groups. In this analysis we found that the group in which significant pseudoword EEG responses were observed also had significantly larger behavioral effects in the target detection task (Figure 7, bottom panel). Specifically, this group had larger differences in RTs between targets occurring in the 3rd position of pseudowords vs. targets within non-words [*t*(14) = 2.15, *p* = 0.03; BF_10_ = 4.18 (moderate support)]. However, this effect was not significant for hit rates in the target detection task [*t*(10) = −1.02, *p* = 0.17] (Figure 7, middle panel) or for performance on the 2AFC task [*t*(21) = −0.19, *p* = 0.85] (Figure 7, top panel).

## DISCUSSION

In this study, we tested the sensitivity of the EEG frequency-tagging approach as an online measure for assessing auditory statistical learning of an artificial language, at both the group level and within individual participants. We found that, even after controlling for potential acoustic contributions to the pseudoword frequency, there is still a significant difference between the artificial language and the baseline conditions at the group level. This effect manifested most robustly at the 3rd pseudoword-level harmonic (5.33 Hz), and less reliably at the pseudoword-level rate itself (1.33 Hz). The previously reported decrease at the 4-Hz syllable level for artificial language stimuli was also observed here, but again with low statistical reliability. Effects were observed already during the first exposure block, (3.22 min) and did not change significantly with additional exposure. These results help validate the use of the frequency-tagging approach for assessing SL *at the group level*, while highlighting important considerations for implementing this technique in future studies.

However, at the *level of individual participants*, only 30% showed significant effects of SL in their neural response, and among them the effects did not occur consistently at the same frequencies/harmonics. Conversely, performance on the implicit target detection task administered post-exposure demonstrates that SL occurred in a substantially larger proportion of individuals (70%). Hence, the current results suggest that the EEG-based metric has a lower sensitivity than some implicit behavioral metrics and likely underestimates the prevalence of SL in individual participants.

### Strengths and Weaknesses of the Frequency-Tagging Approach for Assessing SL

The EEG frequency-tagging approach has been proposed as a more direct means for assessing SL, circumventing the need for behavioral post-exposure testing. Among its strengths is its online nature, which allows researchers to track the formation of a neural representation for pseudowords over time, without introducing a dual task. This approach has been successfully applied for studying neural processing of familiar and unfamiliar languages, and how the representation of different linguistic levels of speech is modulated by factors such as attention, state of arousal, and consciousness (Chen et al., 2020; Ding et al., 2016; Getz et al., 2018; Har-shai Yahav & Zion Golumbic, 2021; Luo & Ding, 2020; Makov et al., 2017; Niesen et al., 2019). The frequency-tagging approach has also brought great excitement to the field of statistical learning, since it offers a way to dissociate between the acoustic-representation of individual elements in a stream (e.g., syllable rate; 4 Hz in the current study) and its parsing into larger units (e.g., pseudoword rate; 1.33 Hz and its harmonics in the current study) that reflects higher-level generalization and learning (Batterink & Paller, 2017, 2019; Buiatti et al., 2009; Elmer et al., 2021; Getz et al., 2018; Henin et al., 2021).

However, here it is crucial to note an important methodological caveat: The interpretation that peaks in the neural response at the pseudoword rate reflect detection and parsing of pseudowords relies on the assumption that these peaks cannot be derived from the acoustics of the stimulus alone. Unfortunately, this assumption does not seem to hold for the type of stimuli typically used in the triplet-based artificial language SL paradigm. As shown in the modulation spectra when testing several different combinations of triplet syllables (Figure 1), a prominent peak can be seen at the triplet rate in addition to the syllable rate. This peak is generated due to subtle yet systematic differences in the envelope shape of different syllables, which are presented consistently at the same position—an inherent feature of pseudowords. These caveats of the frequency-tagging approach have recently been pointed out when using bisyllabic words in real languages (Har-shai Yahav & Zion Golumbic, 2021; Luo & Ding, 2020). Similarly, for artificial languages, the elegant re-analysis of the data in Batterink and Paller (2017) showed that at least part of the neural response at the triplet-rate frequency can be attributed to differences in the OCP of different syllables rather than SL per se (van der Wulp, 2021). Therefore, in order to avoid overinterpretation of these peaks, adequate controls must be implemented in all studies.

Here we addressed this concern by introducing a position-controlled baseline stimulus, which shared the same modulation spectrum as the artificial language stimulus. As expected, in addition to the neural response at the syllable rate, the response to this position-controlled stimulus contained a prominent peak at the triplet rate and its harmonics, even though it contained no statistical regularities. This demonstrates the methodological caveat of frequency-tagging mentioned above—that the mere existence of a triplet-rate peak is not, in and of itself, an indication of statistical learning. Nonetheless, when comparing the neural response to the two stimuli at the group level, the triplet-rate peak (and its harmonics) was significantly larger in response to the artificial language stream relative to its position-controlled baseline stimulus. This pattern suggests that the neural response at the triplet rate and its harmonics reflects *a combination* of acoustic responses as well as responses reflecting detection of the underlying statistical structure and/or pseudoword boundaries.

Interestingly, the strongest effect was not found at 1.33 Hz, which is the triplet rate itself, but rather at its 3rd harmonic (5.33 Hz). This is similar to the pattern reported by a recent electrocorticography (ECoG) study, where the most prominent effects of SL were also found at harmonics of the triplet rate (Henin et al., 2021). Moreover, as detailed below, when inspecting the individual-level spectra, we found great variability in which frequencies showed the most prominent SL effects. The manifestation of effects at harmonic frequencies is a natural consequence of presenting rhythmic stimuli, and should not necessarily be interpreted as carrying nuanced information regarding the nature of neural encoding for these stimuli (Zhou et al., 2016). However, this variability does present another potential caveat for the utility of the frequency-tagging approach.

### Assessing SL in Individual Participants

One of the main goals of the current study was to investigate the sensitivity of different measures of SL at the level of individual participants. Due to the proposed ubiquitous nature of SL and its proposed importance for language acquisition, we expected to find evidence for SL in most participants. However, this was not case. Rather, the pattern emerging from comparing the three independent measures used here—the explicit 2AFC, the implicit target detection task, and the frequency-tagged EEG spectrum—illustrates the operational challenge of empirical assessment of SL. The 2AFC test failed to show a significant effect at the group level, and at the individual level only 3 participants (7.5%) showed significant effects. These poor performance levels are in line with previous studies where reported group-level detection rates range between 54% and 74%, and individual-level significance rates are low (fewer than 50% of participants; Franco, Gaillard, et al., 2015). This task also has been shown to have a medium-low test-retest reliability (Erickson et al., 2016; Siegelman & Frost, 2015), and several methodological factors have been proposed explaining the low sensitivity of the 2AFC approach (Siegelman et al., 2017). There also seems to be a lack of correlation among various auditory SL tasks themselves. A study comparing several auditory SL paradigms using the explicit 2AFC task on the same participants reported a lack of correlations between these very similar paradigms that only differed in the language that was used (Erickson et al., 2016). The authors therefore concluded that these low correlations were most likely the result of the poor psychometric properties of the 2AFC measure and that using a composite score of all these measures combined gives the clearest picture of the situation. Given these low performance rates, which do not coincide with other measures, it seems that the 2AFC metric is not sufficiently reliable for determining whether SL has or has not occurred in individual participants.

The weakness of explicit 2AFC testing has led to the development of more implicit measures for assessing statistical learning. Some examples of implicit tasks include the target detection task (Batterink & Paller, 2017; Batterink et al., 2015) adapted in the current study, as well as rapid serial auditory presentation (Franco, Eberlen, et al., 2015), statistically induced chunking recall (Isbilen et al., 2017, 2020), and the click detection task (Franco, Gaillard, et al., 2015; Gómez et al., 2011). These tasks all rely on a similar principle: If pseudowords in the stream are learned, this will produce a faster implicit response to targets that are associated with that pseudoword.

In the current study, the implicit target detection test showed evidence for SL in the largest proportion of participants, with 18/27 participants (70%) showing a significant effect on *either* hit rate or RT. Indeed, of all the measures tested here, the implicit task seemed to be the most sensitive to SL at the individual level. At the same time, this measure is also not ideal. Since only 3 participants showed significant effects in *both* RT and hit rate, perhaps due to speed-accuracy tradeoffs, this dilutes the group-level effect of both measures and maintains the operational ambiguity as to which is the “best” measure to use. This ambiguity is mirrored when looking at previous studies that employed implicit behavioral tasks and report a highly variable proportion of effects in individual participants. For example, Batterink et al. (2015) report SL effects in 43% of participants using a task similar to the one used here, Gómez et al. (2011) reported SL effects in 85% of participants, whereas Franco, Eberlen, et al. (2015) found these in only 35% of participants, with many participants actually showing reverse effects. Moreover, as has been pointed out previously, the implicit nature of the task makes it difficult to ascertain whether significant effects truly reflect lexical detection of newly learned words, or if effects are driven by lower level perceptual familiarity with syllable combinations (Batterink et al., 2015; Franco, Eberlen, et al., 2015; Isbilen et al., 2017). Moreover, in the current study, the implicit task was always administered after the explicit 2AFC task, which may have reinforced previous learning due to the additional exposure to the pseudoword syllable combinations (although in the 2AFC task participants were also exposed to part-words and were not given feedback regarding their performance). Taken together, although in the current study the implicit target detection task seemed to be in line with the proposed ubiquitous nature of SL, the large variability across behavioral studies (in methods and results) makes it difficult to wholeheartedly accept these implicit measures as a reliable benchmark for assessing SL. Further, the cross-study discrepancies make it extremely difficult to determine the true extent of SL in individual participants.

The diverse and inconclusive nature of indirect behavioral measures was one of the primary motivators for looking to neural measures as more direct signatures of SL. The current study is the first to assess the robustness of neural SL measures in individual participants using the frequency-tagging approach. In contrast to the expected ubiquity, we found that only 12/39 participants (30%) showed significant effects of SL in their EEG spectra. One reason for this might be the poor SNR in individual-level scalp level EEG, which might be improved upon using other neurophysiological measures. For example, a recent ECoG study, which by its nature is based on individual participants, was able to demonstrate robust neural response at pseudoword-related frequencies, suggesting that improving the SNR might lead to more robust results (Henin et al., 2021). However, another factor that exacerbates the complexity of interpreting the frequency-tagging results is that the effects of SL were not observed consistently at the same frequencies, but rather were seen at different harmonics of the pseudoword rate across participants. This was also the case in the ECoG data reported by Henin et al. (2021), which leaves many questions open regarding the underlying mechanism driving these spectral modulations. We can hope that future methodological advances will improve the SNR of frequency-tagging measures, which in turn might reveal more extensive evidence for SL. However, at present, the current results leave us wondering whether the low prevalence of neural effects corresponding to SL are merely a result of poor SNR or if they challenge the assumption of the ubiquitous nature of SL. Our results are of particular importance for endeavors to assess the “cognitive state” of unresponsive patients, using scalp EEG (Gui et al., 2020; Sokoliuk et al., 2021).

In the absence of a gold standard indication for SL, we turn to look for evidence of converging operations among the multitude of tests that all supposedly measure whether SL has taken place. Unfortunately, results from the different behavioral and neural measures do not seem to converge as one might expect if they truly capture the same cognitive operation. In testing whether neural results corresponded in any way with the behavioral responses, we found that the subgroup of participants who showed neural evidence for SL also had slightly faster RTs in the implicit target detection task than those who did not. However, no correspondence was found when examining the within-participant correlation, nor were there any correlations with other behavioral measures. The current results align with previous studies that also reported no correlation between results on explicit and implicit methods of testing for SL (Batterink et al., 2015; Franco, Eberlen, et al., 2015; Isbilen et al., 2020; Misyak et al., 2010). In the few studies where there were significant correlations between explicit and implicit measures, these were not consistent across different modalities (Isbilen et al., 2020), or differences in the explicit task (Batterink & Paller, 2017). Some have opted to interpret the lack of a reliable cross-measure correlation as an indication that each measure picks up on a different cognitive aspect of SL, for example, suggesting a dissociation between explicit recall and implicit learning (Batterink et al., 2015; Franco, Eberlen, et al., 2015; Isbilen et al., 2017). This debate in the literature is ongoing and there does not seem to be a consensus about whether these measures reflect the same processes. The results of the current study do not attempt to answer this question, but rather address the possibility that we cannot rule out that all of these measures—behavioral and neural alike—are simply too crude or too indirect for assessing the formation of internal memory representations arising from SL. Consequently, it seems that we still lack a “ground truth” indication for SL, which (at the moment) severely limits the extent to which this ability can be studied at the level of individual participants.

### Conclusions

The current study highlights the utility and the limitations of the EEG frequency-tagging approach as a research tool for studying SL. At the group level, our results indicate that even after controlling for possible acoustic confounds, peaks in the neural signal at the pseudoword frequency (and its harmonics) likely reflect the implicit detection of underlying transitional probabilities between syllable triplets. However, our data also suggest that the frequency-tagging approach might not be as useful for studying SL in individual participants. The frequency-tagged EEG data were less sensitive to SL than the implicit behavioral test, with effects manifesting at different frequencies across participants. Moreover, the overall low correspondence between the different behavioral and neural metrics, which supposedly all test for SL, leaves much to be desired in our quest to identify the best operationalization for studying SL. Whether the low-reliability of the EEG results is due to the low SNR of this tool or whether it is indicative of a deeper flaw in the frequency-tagging approach, is beyond the scope of this paper. Therefore, while some researchers may find this experimental approach suitable for their needs, the limitations and potential confounds highlighted here should be taken into consideration when interpreting and comparing results across studies, particularly regarding individual differences.

## FUNDING INFORMATION

Elana Zion Golumbic, German-Israeli Foundation for Scientific Research and Development (https://dx.doi.org/10.13039/501100001736), Award ID: 1422.

## AUTHOR CONTRIBUTIONS

**Danna Pinto**: Data curation: Lead; Formal analysis: Equal; Investigation: Equal; Methodology: Equal; Writing – original draft: Equal; Writing – review & editing: Equal. **Anat Prior**: Conceptualization: Equal; Methodology: Supporting; Writing – review & editing: Equal. **Elana Zion Golumbic**: Conceptualization: Equal; Data curation: Equal; Formal analysis: Equal; Funding acquisition: Lead; Investigation: Equal; Methodology: Equal; Project administration: Lead; Resources: Lead; Supervision: Lead; Validation: Lead; Writing – original draft: Equal; Writing – review & editing: Equal.

## TECHNICAL TERMS

Transitional probability: The statistical relationship between two consecutive events.
Pseudowords: Combinations of syllables that are not lexical entities and do not carry semantic meaning in a particular language.
Frequency-tagging: A method by which specific features of a stimulus are presented at a particular frequency, which allows discerning a neural-signature of this feature in the spectrum of the neural data.
Obligatory contour principle (OCP): A hypothesis in phonology which states that certain phonetic features may not occur consecutively.
Modulation spectrum: The frequency content of the envelope of a particular signal (for example, of the speech-envelope).
Inter-trial phase coherence (ITPC): A measure of phase consistency across trials.
