# Supplementary material for: Assessing the Sensitivity of EEG-Based Frequency-Tagging as a Metric for Statistical Learning
Source: Neurobiol Lang (Camb). 2022 Feb 16;3(2):214–34. doi: 10.1162/nol_a_00061 (PMC10158570; doi:10.1162/nol_a_00061)
Supplement: Supplementary file 1 [file nol-3-2-214-s001.docx]

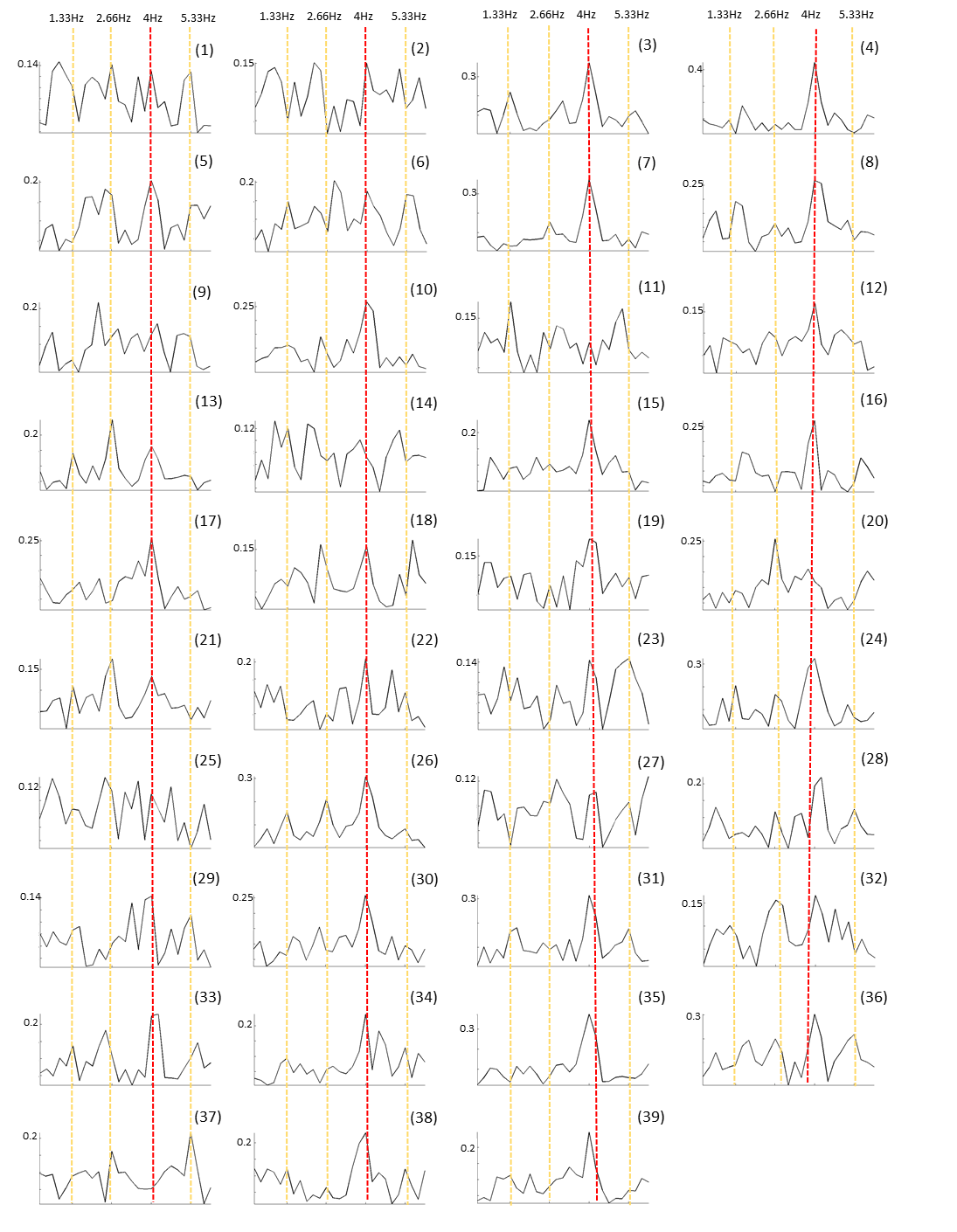


**Figure S1. Individual ITPC spectra - Position-Controlled Baseline condition.** Neural ITPC spectra of all 39 participants in the Position-Controlled Baseline Condition, averaged over all electrodes. The dashed red line indicates the syllable-rate (4Hz) and the yellow dashed lines indicate the triplet frequency (1.33Hz) and it’s 1^st^ and 3^rd^ harmonic (2.66Hz and 5.33Hz, respectively). ITPC plots are scaled individually, with the participant number shown in parentheses on the top-left. As expected, most participants show a peak at the syllable rate. At the same time, the magnitude and frequency-specificity of this peak differs somewhat between individuals, demonstrating the variability of this measure. In addition, many participants have peaks at frequencies associated with syllable triplets, despite the fact that transitional probabilities between all syllables was constant (p=0.2). This suggests that presenting different sets of syllables at each position can generate peaks in the ITPC spectra, likely due to differences in their temporal contour. Therefore, the presence of such peaks, in and of themselves, should not necessarily be taken as evidence for detection of statistical regularities between syllables.
